# Curcumin protects SH-SY5Y cells from oxidative stress by up-regulating HO-1 via Phosphatidylinositol 3 Kinase/Akt/Nrf-2 and down-regulating HO-2

**DOI:** 10.1186/1750-1326-7-S1-S14

**Published:** 2012-02-07

**Authors:** Wenke Yin, Xiong Zhang, Xiaodong Shi, Yu Li

**Affiliations:** 1 Department of Pathology, Chongqing, Chongqing Medical University, 400016, China; 2 Chongqing Key Laboratory of Neurobiology, Chongqing Medical University, Chongqing, 400016, China; 3 Institute of Neuroscience, Chongqing Medical University, Yuzhong District Yuanjiagang Yixueyuan Road No.1, Chongqing, 400016, China

## Background

Oxidative stress is considered to have a causative role in the development of central nervous system diseases, such as Alzheimer’s disease, Parkinson’s disease, tumor, etc. Curcumin, a polyphenol extracted from rhizomes of the plant Curcuma longa, is widely reported to have diverse anti-oxidative stress effects, but the underlying mechanism has not been fully elucidated. We investigated the mechanism underlying the neuroprotective properties of curcumin in human neuroblastoma SH-SY5Y cells subjected to oxidative stress.

## Methods

The cells were treated with curcumin at different concentration (0, 1.25, 5, 20 μM) for 24, 48, 72 h, and/or with PI3K inhibitor LY294002 or Nrf-2 siRNA, the concentration- and time-dependent protection of curcumin against H_2_O_2_-induced toxicity was measured as ROS production and reduced cell growth. RT-PCR and Western blot were applied for detecting the expression of HO-1, HO-2, PI3K, AKT and Nrf-2 at mRNA and protein levels, also, the production of ferritin was measured by WB.

## Results

The expression of HO-1 and the production of ferritin were significantly increased, but the expression of HO-2 was decreased. Furthermore, curcumin could significantly induce the protein and mRNA expression of PI3K, AKT and Nrf-2 (*P*<0.05), while the protective effects of curcumin were prevented by PI3K inhibitor LY294002 or Nrf-2 siRNA.

## Conclusion

Taken together, the data show that the cytoprotection of curcumin against oxidative stress in SH-SY5Y cells is by up-regulating HO-1 expression via PI3K/AKT/Nrf-2 intracellular signaling pathway and down-regulating HO-2 expression. Which suggests that curcumin is beneficial in the prevention and treatment of some CNS disease.

